# Using electrophysiological measures to evaluate the sense of presence in immersive virtual environments: An event‐related potential study

**DOI:** 10.1002/brb3.2269

**Published:** 2021-06-26

**Authors:** Simone Grassini, Karin Laumann, Sebastian Thorp, Virginia de Martin Topranin

**Affiliations:** ^1^ Department of Psychology Norwegian University of Science and Technology Trondheim Norway; ^2^ Department of Circulation and Medical Imaging Norwegian University of Science and Technology Trondheim Norway

**Keywords:** electroencephalogram, event‐related potential, sense of presence, user experience

## Abstract

Sense of presence has been often explored in the context of virtual reality (VR) and immersive visual technologies; however, standardized and objective measures of the sense of presence have been difficult to find. Studies attempting to find physiological correlates of sense presence using electroencephalography (EEG) have reported mixed results. In the present study, we used brain event‐related potentials (ERPs) elicited by auditory stimuli to identify an objective physiological index of sense of presence during VR, attempting to replicate the findings of previous studies and explain the heterogeneity of results reported in the literature. Participants in our experiment were asked to experience an immersive virtual environment using a modern head‐mounted display while passively hearing task‐irrelevant frequent standard and infrequent deviant tones as in a classic auditory oddball paradigm. Subsequently, they were asked to complete a battery of questionnaires aimed to estimate their sense of presence during the VR. EEG and questionnaire data from three‐seventh participants were analyzed. ERP components evoked by the auditory stimuli were then analyzed. Late ERP components (after 450 ms from stimulus onset) registered over central brain areas were associated with the sense of presence as measured with questionnaires, while earlier components were not associated with presence. The use of different questionnaires and the content of the VR environment may both be a plausible explanation for heterogeneous results as reported in previous studies. The present study showed that late ERP components recorded over the central brain may represent good electrophysiological correlates of the subjective sense of presence.

## Graphical abstract

We validated a method to indirectly index sense of presence using EEG. We found linear association between event‐related potentials to task‐irrelevant stimuli during the VR simulation, and reported self‐presence. The present finding will help to establish new methods to objectively evaluate sense of presence.

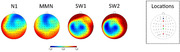

## 1 INTRODUCTION

The surge of interest in virtual experiences along with the technological advancements of a modern head‐mounted display (HMDs) has increased the availability and the quality of VR experiences. Modern immersive systems generally consist of wearable displays (HMDs) and handheld controllers, allowing users to visualize and interact with a simulated environment. The headset provides computerized images on two screens, one for the view of each eye (Slater & Sanchez‐Vives, 2016). Displaying the images using this technique allows the environment's depth to be presented realistically (Urey et al., 2011). These technological developments are likely to have enhanced the sense of presence during the VR experience, which is a crucial factor in the exposure to the simulated environment (Aymerich‐Franch, 2010; Nacke et al., 2010).

Sense of presence has often been associated with the quality of user experience (see e.g., Brade et al., 2017; Busch et al., 2014). The phenomenon has been extensively studied within the frameworks of both theoretical philosophy and empirical research. The importance of understanding and measuring sense of presence is also notable from a practical perspective as a relationship between sense of presence and human performance has been supported by empirical research (Baumgartner et al., 2006; Baus & Bouchard, 2017; Grassini et al., 2021a, but see e.g., Makransky et al., 2019). Sense of presence can be defined as the subjective feeling of “being there” in a computerized environment (see Riva et al., 2003; Slater et al., 1995; Witmer & Singer, 1998). When the sense of presence is high, the virtual nature of a computer‐mediated environment is unnoticed, and users subjectively perceive the simulation as the most relevant reality (Barfield et al., 1995). Level of stimulation, as well as the interactivity of a system (Steuer, 1992), have been proposed to stimulate the human sense of presence.

Several different questionnaires have been developed based on different theoretical frameworks (one of the most prominent conceptualization is the one proposed by Lee, 2004b), and there is no consensus among scholars about which sense of presence construct or which questionnaire is most suitable to measure the phenomenon (Grassini & Laumann, 2020a; Nordin et al., 2014). Moreover, retrospective questionnaires are unable to provide a continuous evaluation of the user experience.

Physiological measures may offer the possibility to assess user experience during virtual reality (VR) experiences in a continuous, direct, and non‐invasive way. Finding a continuous and objective measure of sense of presence would allow researchers to better understand the development of the sense of presence over time and facilitate the quantification of the quality of experience of VR users. Furthermore, this measure would enable researchers to identify strategies to positively modulate the sense of presence, possibly increasing the quality of experience provided by the VR systems and promoting their acceptability (Sadowski & Stanney, 2002). Brain measures such as functional magnetic resonance (Hoffman et al., 2003) and electroencephalography (EEG; Grassini & Laumann, 2020a) are physiological measures that provide a continuous assessment throughout VR exposure. Among brain‐related measures, EEG has found the most use in the study of the sense of presence (Grassini & Laumann, 2020). An EEG system measures the electrical activity of the brain produced by neurons firing simultaneously. This is responsible for an electrical potential significant enough to be measurable by a sensor placed on the scalp (Breedlove & Watson, 2013). Event‐related potentials (ERPs) are brain waves generated in response to stimulation (e.g., an event), averaged across many trials to obtain a reliable estimate of a brain activity that is time‐locked and evoked by a sensory stimulus (Andreassi, 2007). ERP components have been studied as indexes for the allocation of cognitive and attentional resources (Kok, 1997; Luck et al., 2000). One of the most frequently used methodologies for studying the allocation of attentional resources is the dual‐task paradigm (Karatekin et al., 2004). Such experimental paradigm involves the participant performing a primary and secondary task at the same time (Gosselin & Gagné, 2010). The performance of the primary task then utilizes the required attentional resources, while the secondary task depends on the remaining resources. A classic example of such a technique is the auditory oddball paradigm. In this experimental paradigm, the participant is presented with frequent and infrequent “deviant” auditory stimuli continuously presented in random series (Strüber & Polich, 2002). The task is generally implemented as a secondary probing task, and discrimination between frequent and deviant sounds is assumed to absorb only those attentional resources not devoted to the primary task (Kok, 1997). Thus far, few investigations have explored the possibility of using task‐irrelevant ERPs as an index of sense of presence (Burns & Fairclough, 2015; Kober & Neuper, 2012; Terkildsen & Makransky, 2019). These studies investigated the use of ERPs related to resource allocation toward task‐irrelevant tones as an indirect physiological correlate of sense of presence, yielding inconsistent results. This could be due to the allocation of participants into low and high sense of presence groups based on different questionnaires: Short Feedback Questionnaire (SFQ; Kizony et al., 2006), Immersive Experience Questionnaire (Jennett et al., 2008), and Multimodal Presence Scale (MPS; Makransky et al., 2017). Furthermore, all the previous studies had a similar limitation: participants all experienced a slightly different simulated scenario (as they were asked to actively move in the VR), which may have modulated sense of presence along with other factors, such as emotion (especially in the highly emotional game used by Terkildsen & Makransky, 2019).

The goals of the present investigation are to use EEG (1) to find evidence of an association with a subjective sense of presence for one or more electrophysiological indexes and (2) to attempt to explain mixed results as reported in previous EEG studies (Burns & Fairclough, 2015; Kober & Neuper, 2012; Terkildsen & Makransky, 2019), considering potential differences stemming from different questionnaires. To meet these goals, two research questions are investigated in this study: (1) Can electrophysiological data (i.e., from EEG) be used to objectively quantify the sense of presence? (2) Do differences in questionnaires explain the heterogeneity of previous findings (in the studies of Burns & Fairclough, 2015; Kober & Neuper, 2012; Terkildsen & Makransky, 2019)? The experimental framework utilized was the same as that of some previous studies (Kober & Neuper, 2012; Terkildsen & Makransky, 2019). The time windows in which these components were selected—N1 (100–200 s), mismatch negativity (MMN, 150–200 ms), early slow waves (SW1, 400–650 s), and late slow waves (SW2, 600–900 ms)—were analyzed in line with the time windows in which these components were identified in Terkildsen and Makransky (2019). A VR scenario that was passively viewed by participants (roller coaster virtual ride), with no tasks to be performed, was used to determine whether SWs were related to difficulties or workload and to control for possible confounding variables, ensuring the consistency of the experience between participants. The use of different questionnaires to assess sense of presence (Presence Questionnaire (PQ), Slater–Usoh–Steed (SUS), MPS (spatial and self‐presence sub‐categories)) attempted to explain the heterogeneity of results reported in previous investigations. We hypothesized that the heterogeneity of results reported in previous investigations may be due to the different questionnaires assessing the sense of presence that was used for the high and low sense of presence group split and/or as a limitation and possibly a problem with the use of the median split as a grouping strategy.

## 2 METHODS

### 2.1 Participants

Participants were 40 undergraduate students, recruited among volunteers in the student population at the Norwegian University of Science and Technology (Trondheim campuses). The participants were reported to be generally healthy. The study was conducted with the understanding and written consent of each participant and in accordance with the Declaration of Helsinki. The experimental protocol was notified and approved by the Norwegian Centre for Research Data (NSD) prior to data collection. All participants were required to be proficient in English, and all the materials used in this study were in English. It was also a prerequisite that the participants declared to have a normal (or corrected‐to‐normal) vision. All the participants declared to have no diagnosis of psychiatric disorders and/or history of epileptic seizures or current intake of psychotropic drugs. None of the participants declared to be familiar with the game prior to the experiment.

From the initial 40 participants, three of them were eliminated from any analysis. Two of them had their data compromised by technical problems (very short experimental sessions recorded). One of them was excluded as it asked to withdraw from the experiment due to excessive simulation sickness after only 2 min of the experiment. The analyses were based on the data from the 37 remaining participants (15 males and 22 females, mean age of 23.97 years SD = 3.37). The final number of participants in our study was in line with one of previous similar studies (Burns & Fairclough, 2015, *N* = 20; Kober & Neuper, 2012, *N* = 40; Terkildsen & Makransky, 2019, *N* = 34).

### 2.2 VR scenario

**FIGURE 1:**
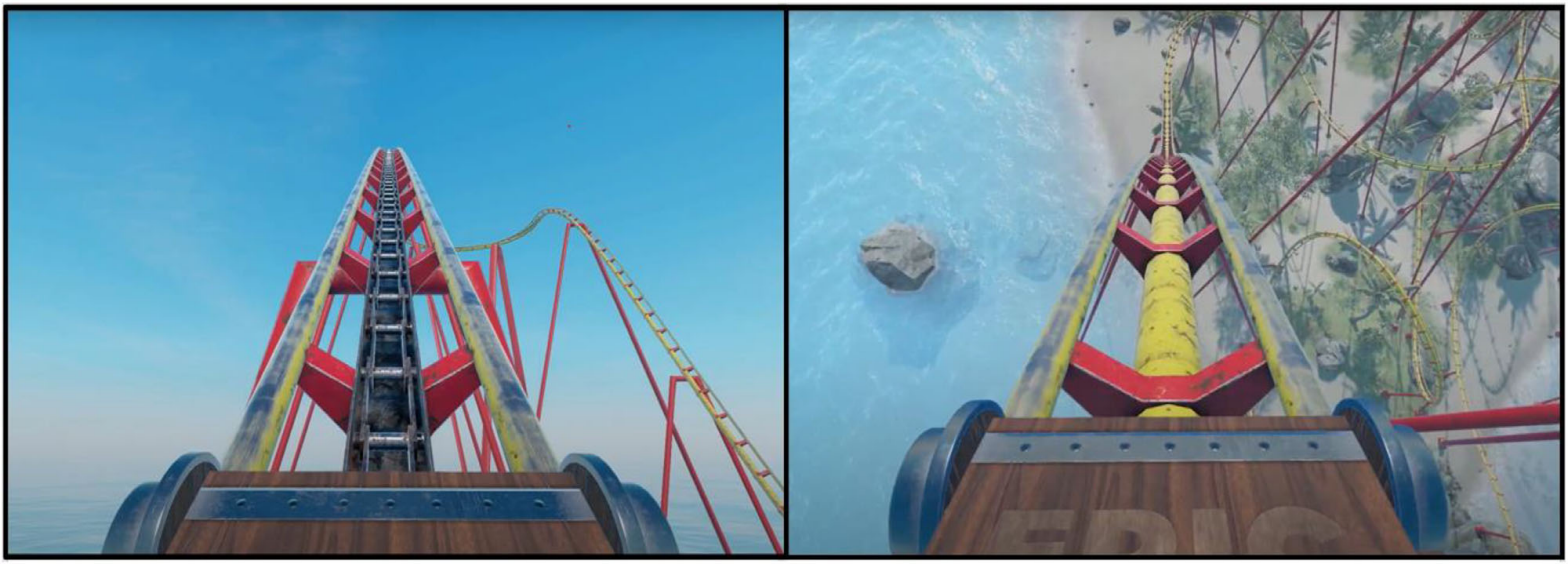
Example images from “Epic Roller Coaster” by B4T Games, scenario “tropic island”; reproduced with permission

The VR scene presented a first‐person roller‐coaster ride. The ride is part of the selection of rides available in a customer‐oriented videogame (“Epic Roller Coaster” B4T games). Such type of VR was chosen for the ability to induce, even after short use, a high sense of presence as previously shown (see e.g., Grassini et al., 2021a; Grassini et al., 2021b). The virtual ride was set in a fictitious tropic island environment. Examples from the scenes are presented in Figure 1. The VR was delivered using the Oculus GO HMD (Facebook Technologies), which features a 5.5‐inch Liquid Crystal Display with a 2560 × 1440 (1280 × 1440 pixels per eye) resolution, a refresh rate of 72 or 60Hz (depending on the application running), and an field of view of 101 degrees (see the producer website at http://www.oculus.com/go/). The developer and copyright holder of the software gave consent for the use of the game as well as for disclosing images of it in activities aimed at scientific communications.

### 2.3 Task‐irrelevant stimulus

For 2 min before the start of the experiment and for the entirety of the virtual roller‐coaster ride session, task‐irrelevant tones were played via two speakers (Logi Multimedia Z200) situated 45 cm behind the subject's position at 20 cm to each other. In line with the argument of Terkildsen and Makransky (2019), speakers were used instead of headphones as the tones of the secondary task were supposed to feel external from the simulation and separated from the VR scenes. The participants were asked not to react to the tones, and they were told that the tones were external of the VR environments and were playing as part of the experiment. The tones were played for 150 ms at a volume of an average of 52 decibels, as measured from the subject's position, with an inter‐stimulus interval of 1 s. The tones were randomized between a standard 1200 Hz tone (probability of 0.8) and a deviant 2000 Hz tone (probability of 0.2). The utilized script excluded the possibility for two subsequent deviant tones (as in Terkildsen & Makransky, 2019). The experimental paradigm was developed following the methods used in Kober and Neuper (2012) and Terkildsen and Makransky (2019) and adapting it to our laboratory setting, avoiding critical changes. The experimental paradigm was programmed and run using OpenSesame 3.2 (Mathôt et al., 2012).

### 2.4 Procedure

After giving written consent, the participant entered the laboratory, sat in a chair, and was fitted with the EEG electrode cap equipment and the VR headset. Prior to the start of the VR scenario, 2 min task‐irrelevant ERP stimuli were played to habituate the subjects to the occurrence of the background sound. As we did not have any a priori hypothesis on brain activity during this pre‐exposure time, EEG data was not recorded until the beginning of the VR stimulation.

The subjects were instructed to press the start button on the control device, at which point the recording of EEG began. The experimenter exited the experimental room after instructions were provided. The stimulus played for 8 min. At the end of the VR exposure, the EEG cap was removed, and the participant could stand up and exit the testing room. In the adjacent room, subjects were asked to fill out the set of questionnaires. One researcher provided instructions about the questionnaires and was available for questions.

### 2.5 Questionnaires

The pre‐session questionnaire consisted of a consent form, including demographic questions of the participant's age and sex. Furthermore, three different PQs were used. These questionnaires were selected for their wide use in the literature (see e.g. Grassini & Laumann, 2020a), as well as they were used in previous similar studies (Kober & Neuper, 2012; Terkildsen & Makransky, 2019). Questionnaire data were collected using traditional pen‐and‐paper, and sense of PQs were administered in random order.

1. The PQ

The questionnaire is based on the theory of presence of Witmer and Singer (1998). According to this theory, sense of presence is a construct consisting of three factors: The degree to which the VE is experienced as the dominant reality, the sense of being in the VE, and the degree to which individuals view the VE as a dimension they visited rather than just images on a screen. This version of the PQ questionnaire consists of 32 items on a Likert scale ranging from 1 (not compelling) to 7 (very compelling). The participants were asked to answer the items by placing an “X” in the appropriate box. The survey consisted of items like “How inconsistent or disconnected was the information coming from your various senses” (Witmer & Singer, 1998). Research by the authors indicated that internal consistency measures of reliability were reported to be good at 0.81 (Cronbach's alpha). Some of the items of the PQ questionnaire were not relevant for our VR scene (items number 1, 13, 17, 19, 20, 21, 24, 25, 29, 30, 31; e.g., the ones relative to the ability to control the events as “Were you able to anticipate what would happen next in response to the actions that you performed” or “How well could you actively survey or search the virtual environment using touch,” see Witmer & Singer, 1998, pp. 232–233).

2. The SUS.

The SUS consists of six items that are scored by the participant on a 7 point‐Likert scale, where 7 represents the highest level of sense of presence. In creating the scale, the authors proposed that sense of presence is both an outcome of internal (user) and external (system) factors (Slater et al., 1994). External factors were identified using existing research and included variables like the resolution of the display and interactivity with the environment. Internal factors were identified based on primary human senses like vision, kinetics and auditory, and perceptual position (von Baren & Ijsselsteijn, 2004). Based on this conceptualization, an empirical model was presented. From this internal/external framework, three conceptual categories of sense of presence were proposed: the degree to which the virtual environment becomes the dominant reality, the subjective feeling of being in the virtual environment, and the degree to which the virtual environment is remembered as a distinct location. The original questionnaire consisted of three items but has since been extended to six items.

3. MPS

The MPS, developed by Makransky et al. (2017), was chosen as it was used in Terkildsen and Makransky (2019) and was created to be optimally adaptable for measuring the sense of presence in modern VR. The questionnaire consists of 15 items, providing a subjective measure of sense of presence pertaining to stimulations in VR. The MPS is based on Lee's (2004b) multi‐dimensional conceptualization of sense of presence, consisting of three underlying dimensions of sense of presence: spatial, social, and self. Each of these dimensions is measured by five items that are all rated on a Likert scale from 1 (strongly agree) to 5 (strongly disagree). Confirmatory factor analysis by Makransky et al. (2017) gave evidence to the three‐dimensionality of the model. Additionally, the results indicated that all items were loaded on a single unidimensional scale. Finally, Cronbach's alpha indicated a good internal reliability (α = .86). As in our experiment, social entities were not presented, the five items related to social presence were not used. The remaining two dimensions of the questionnaire (spatial and self) were analyzed separately in the present article. Hereinafter, these two inventories will be referred to as MPS(sp) and MPS(se), respectively.

### 2.6 EEG recording

EEG signal was recorded and digitalized at 512 Hz, using ANT Neuro EEGO sport amplifier (ANT Neuro), from 64 silver cloride (Ag/AgCl) active electrodes arranged in a 10/20 configuration cap (Wavegaurd, ANT Neuro). CPz was used as a reference electrode, and the ground electrode was placed on AFz. Electrode gel was used to increase the conductivity throughout the experiment, and the impedance levels of each electrode were set below 50 kΩ before the beginning of the experiment.

#### 2.6.1 EEG data pre‐processing

For the ERP analyses, data‐pre‐processing pipeline script was created considering the suggestions of the Makoto's preprocessing pipeline (see Makoto, 2020). EEG data were processed offline using MATLAB (v. R2019b; The MathWorks Inc.) and with the EEGLAB toolbox version 2019.1 (Delorme & Makeig, 2004). Data were high‐pass filtered at 0.1 Hz and low‐pass filtered at 40 Hz using a Hamming windowed‐sinc finite impulse response filter (EEGLAB function “pop_eegfiltnew”), and then down‐sampled to 256 Hz. To ensure that most of the 50 Hz line noise was removed from the data, the CleanLine plugin for EEGlab was used, in the version available in the PREP pipeline plugin v. 0.55.4 (EEGLAB function “cleanLineNoise”; Bifdely‐Shamlo, 2015). Bad channels were individuated and eliminated automatically using channel statistics (channel kurtosis, EEGLAB function “pop_rejchan”). Bad channels were interpolated using spherical interpolation (EEGLAB function “pop_interpolate”). Data were then re‐referenced to average and epoched (−100 to 900 ms from stimulus onset). Segments containing artifacts were rejected first based on voltage threshold (←500 μV, >500 μV EEGLAB function “pop_eegthresh”), and then based on joint probability (6 SD for single channels and 2 SD for all channels; EEGLAB function “pop_jointprob”). The quite lax rejection rate was used to allow the later independent component analysis (ICA) analysis to optimally individuate eye movements and other non‐brain artifacts in the data. The trial rejection rate was aimed at around 10% of the trials. The remaining data were explored with extended Infomax ICA (EEGLAB function “pop_runica.m”) to identify and subsequently attenuate eye‐blink, eye‐movement, heart‐beat artifacts, and other possibly non‐brain activity. ADJUST 1.1.1 (EEGLAB function “ADJUST”; Mognon et al., 2011; Pontifex et al., 2017) was used to identify and eliminate artefactual independent components. The baseline was then removed from the ERPs (–100 to 0 ms).

### 2.7 Data analysis and statistics

After data pre‐processing in Matlab, EEG data was loaded in Brain Vision Analyzed (BVA v. 2.2) for further inspection. ERPs figures and scalp map topographies were plotted using BVA. Based on previous studies ERP components latencies were established. Upon further examination of the ERPs (see Figure 3), the components in our experiment had similar activity patterns as identified in previous studies (Kober & Neuper, 2012; Terkildsen & Makransky, 2019). Therefore, the latencies used for selecting ERP components were the same as in Terkildsen and Makransky (2019), except for N1 (Terkildsen & Makransky, 2019, used 120–200 ms for N1, even though the time window between 100 and 200 ms has been the most commonly used for auditory N1; see e.g., Finnigan et al., 2011; Luck, 2014). The measure of mean area amplitude was calculated averaging the amplitude of the ERP signal for the N1 (100–200 ms), MMN (150–200 ms), SW1 (400–650), and SW2 (650–900 ms) components. MMN was calculated as the amplitude difference between the wave produced by the deviant tone minus the one produced by the frequent tone. The electrodes were selected based on Terkildsen and Makransky (2019) and were the midline electrodes Fz, Cz, and POz.

Statistics were computed using IBM SPSS Statistics v. 26 and Matlab (R2014). To assess the brain activity differences between high and low levels of sense of presence participant groups, univariate repeated measure analysis of variance (ANOVA) 2 × 3 analyses were calculated. The analyses were computed using the sense of presence group as a between‐subject factor (low vs. high sense of presence group) and electrode position (Fz, Cz, POz) as a within‐subject factor. The analyses were performed separately for each ERP component of interest and the different grouping resulted from different questionnaires. When the sphericity assumption was violated (Mauchly's test of sphericity), Greenhouse–Geisser correction was used. Corrected *p*‐values were reported throughout the manuscript, while uncorrected degrees of freedom are reported. Correlation between variables was computed using Pearson's correlation. In the analyses between ERP components and questionnaires scores, possible false positives due to outliers were controlled. For these correlations shown as statistically significant in traditional uncorrected Pearson's analyses, further robust correlation analyses were computed using the Robust Correlation Matlab toolbox (function “skipped_correlation” (see Pernet et al., 2013). The skipped correlation function calculated robust correlations using Pearson's correlation after bivariate outliers were removed. Robust correlation is mathematically based on the data distribution central point (mid‐covariance determinant) and the orthogonal distances for each data point from the data distribution center. Data points located outside the orthogonal limits (calculated using the “idealf” estimator of the interquartile range; see Pernet et al., 2013; Wilcox & Keselman, 2012) were removed and Pearson's *r* calculated. The significance level was estimated based on confidence intervals (95% computed by bootstrapping “nboot = 1000” the data without outliers).

All the statistical tests performed used the significance level of *p* < .05. Standard correlation statistics were one‐tailed, as the direction of the relationship between the variables was deductible from the results reported in previous investigations and from a theoretical point of view. As few variables were examined and all were selected a priori to the investigation to increase comparability with previous studies using a similar experimental design, it was decided not to correct multiple comparisons when analyzing the different questionnaires individually.

## 3 RESULTS

### 3.1 Questionnaires

The results of the questionnaires evaluating the sense of presence were first descriptively analyzed. For the PQ, the mean score was 4 (SD = 0.81), for the SUS the mean was 3.99 (SD = 1.14), for the MPS(sp) the mean was 3.06 (SD = 0.85), and for the MPS(se) the mean was 2.55 (SD = 0.86). These scores are generally in line with the results reported in previous studies (Grassini et al., 2021a; Usoh et al., 2000), however, slightly lower than those reported for the PQ and SUS in the study of Kober and Neuper (2012). For the MPS(sp) and MPS(se), the score of sense of presence was in line with the values reported in Terkildsen and Makransky (2019). The scores of these four inventories were then correlated. Results of the correlations are shown in Table 1 and suggest that the scales are moderately to highly correlated (Mukaka, 2012).

**TABLE 1:** Results of Spearman's correlation, between the four different questionnaires analyzed in the present study

	**Presence Questionnaire (PQ)**	**Slater–Usoh–Steed (SUS)**	**Multimodal Presence Scale‐spatial (MPS(sp))**	**MPS‐self (MPS(se))**
PQ	–	.635***	.585***	.471**
SUS	.635***	–	.737***	.663***
MPS(sp)	.585***	.737***	–	.742***
MPS(se)	.471**	.663***	.742***	–

** *p* < .01.

*** *p* < .001.

**FIGURE 2:**
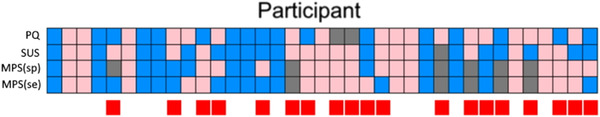
The figure shows individual participants’ (*N* = 37) grouping after the median‐split division based on different questionnaire scores (for Presence Questionnaire (PQ), Slater–Usoh–Steed (SUS), Multimodal Presence Scale‐ spatial (MPS(sp)), and Multimodal Presence Scale‐ spatial‐self (MPS(se)). Blue color indicates participants assigned to the low sense of presence group, pink color indicates participant assigned to the high sense of presence group, and gray color indicate participants assigned to no groups as their questionnaire scores fall on the median score. The red squares on the bottom of the figure indicate those participants that changed the assigned group at least once when evaluating the participant for different questionnaire scores

All the previous investigations associating ERPs with the sense of presence (Kober & Neuper, 2012; Terkildsen & Makransky, 2019) have used the median score of the questionnaire results to divide the participants into high and low‐level sense of presence to allow group comparisons. Hence, we analyzed how the questionnaire used may affect the median split (Figure 2). Nineteen participants out of the 37 analyzed (around 50%) would fall into at least two different groups (between low sense of presence, high sense of presence, median) according to the data of the four different questionnaires employed.

Separately for each questionnaire, the participants displaying questionnaire scores equal to questionnaire overall median scores (gray squares in Figure 2) were not attributed to any group in the analyses. These participants were excluded, independently for each questionnaire, from the successive visualization of the ERPs and statistical analyses. Therefore, the comparison of data involving the low and high sense of presence group contains a different number of participants, depending on the questionnaire used for the median split.

### 3.2 ERPs analyses for high and low sense of presence groups

In order to be able to compare our results with these of previous similar studies, we conducted separate ANOVA analyses, comparing the average amplitudes of the N1, MMN, SW1, and SW2 ERP components, measured from midline electrodes Fz, Cz, and POz, between the low and high sense of presence group. As different questionnaires data attributed participants to different groups, we decided to do separate analyses for each different questionnaire result examined.

To improve the clarity of the figures, in the main body of the article will be visually reported ERPs only relative to the group split obtained using the SUS questionnaire scores. The ERPs based on the SUS questionnaire was chosen for display, as the SUS showed a high correlation with both the MPS questionnaires and the PQ (see Table 1). Furthermore, the SUS is the questionnaire for which the smallest number of participants (1) were removed from the grouping analyses due to participants’ scores falling on the group mean. However, the figures relative to the grouping obtained for all the questionnaires are reported in the Supplementary Data of the present manuscript. Statistical analyses were subsequently performed. Analyses for all the different questionnaires are reported directly in the main body of the manuscript.

**FIGURE 3:**
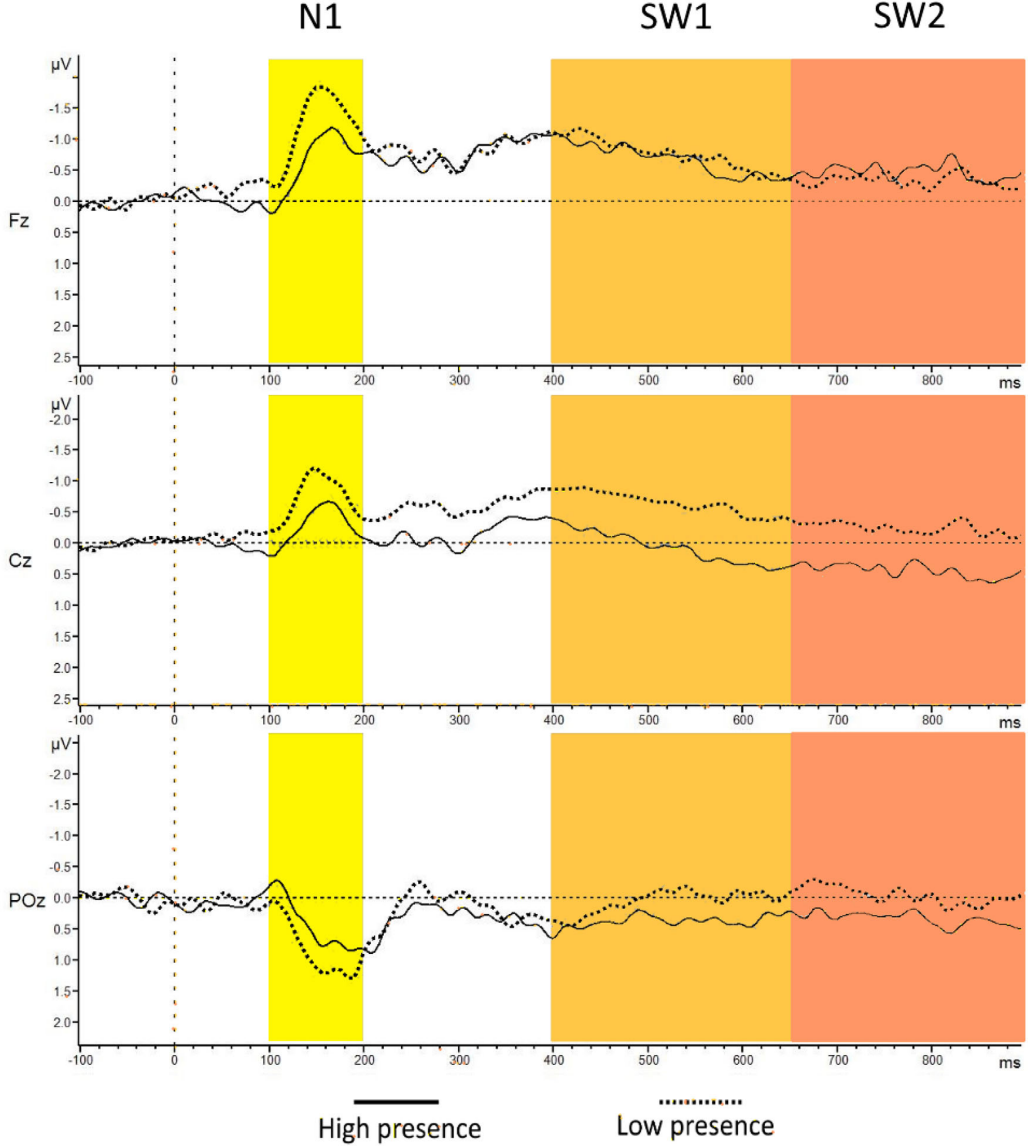
Grand average event‐related potentials (ERPs) for deviant tones recorded in electrodes Fz, Cz, and POz for both groups. The groups were established based on the SUS questionnaire. The highlighted time‐windows indicate the latency windows of interest; N1 (100–200 ms), SW1 (400–650 ms), SW2 (650–900 ms) from the deviant tone onset. Baseline activity is shown (–100 to 0 ms (event onset))

**FIGURE 4:**
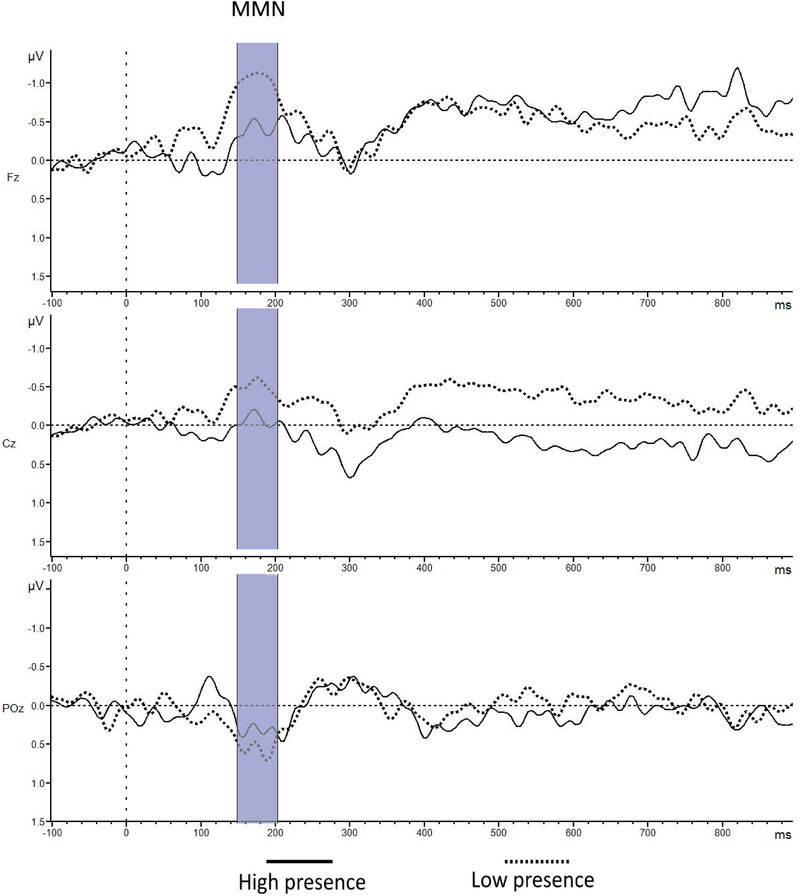
Grand average ERPs with MMN (150–200 ms) component highlighted. The presence groups are estimated based on the SUS questionnaire. The waves were computed subtracting brain activity time‐locked with frequent tones from the one time‐locked with deviant tones, separately for high and low sense of presence groups, established using different questionnaires on sense of presence. Baseline activity is shown (−100 to 0 ms (event onset))

Figure 3 shows the groups’ average ERPs elicited by the deviant tones in the secondary task. In Figure 3 are the ERP components of interest highlighted in yellow, orange, and red color shades. Figure 4 shows the brain waves where the MMN is observable (highlighted in blue). The latter ERPs were obtained subtracting brain activity elicited by the frequent tones from the brain activity elicited by the deviant tones. The mean amplitude, for each one of the selected components, were computed for each participant and each electrode of interest. They were then averaged separately depending on presence group membership and compared. These figures (Figures 3 and 4) comparing high versus low sense of presence groups and based on the median split results from the SUS questionnaire. Analyses for different grouping for the participants, as obtained from the different results of all the analyzed questionnaires, are reported in Supplementary Data (Figures S1 and S2).

**FIGURE 5:**
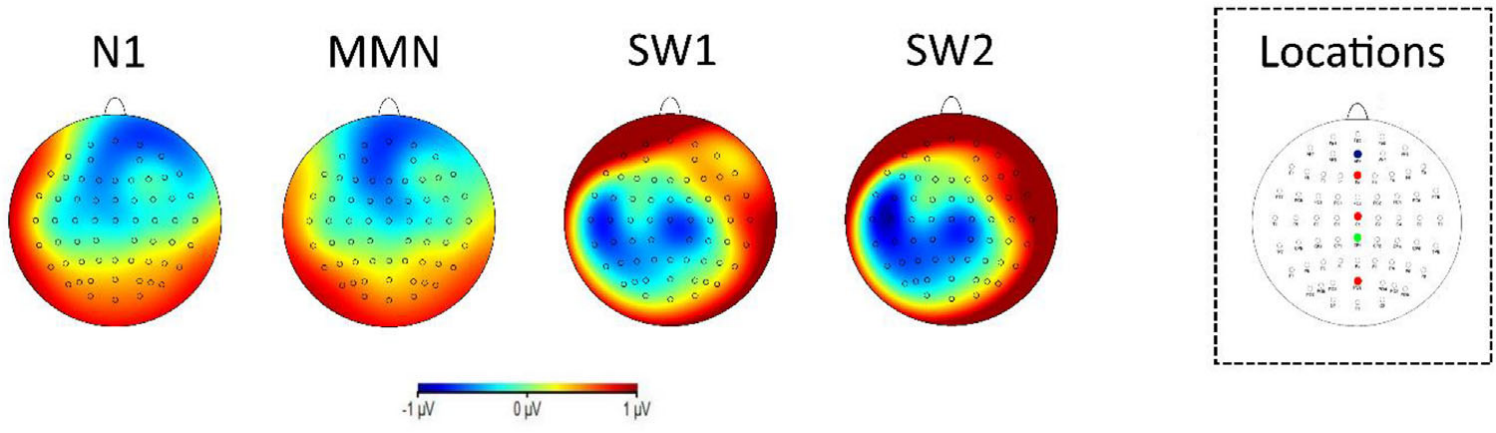
Scalp topographies of the difference of activity (low sense of presence minus high sense of presence), for the ERP components of interest (N1, MMN, SW1, and SW2). On the right is presented the electrode location map. In the template, the electrodes utilized in the ERP analyses are highlighted in red, the reference electrode in green, and the ground electrode in blue

Previous studies have not reported results for activity recorded over lateral brain areas and have reported no hemispheric differences (Kober & Neuper, 2012). As the analyses performed in the present article are theory‐driven (Kober & Neuper, 2012; Terkildsen & Makransky, 2019), analyses on electrodes and brain areas different than the one established a priori (Fz, Cz, POz) were not performed. However, to clarify the overall brain activity related to the studied ERP components, scalp activity topographies for each studied component are presented in Figure 5. These scalp topographies show the brain activity calculated subtracting high sense of presence from low sense of presence grand average ERPs, based on the median split results from the SUS questionnaire.

Repeated measures ANOVA analyses were performed to identify differences in brain activity between the high and low sense of presence group. The statistical results are summarized in Table 2. The high and low sense of presence groups showed no statistically significant differences in their N1 mean area amplitudes, except when the grouping was made using the MPS(sp) questionnaire. The high and low sense of presence group showed no statistically significant differences in their MMN mean area amplitudes, except when grouping was made using the MPS(se) questionnaire. The high and low sense of presence group showed statistically significant differences in SW1 and SW2 mean amplitudes in grouping based on all the questionnaires, with the exception of the one obtained using the MPS(se) questionnaire.

**TABLE 2:** Results of the 2 × 3 univariate repeated measures analysis of variance (*F*‐values). Sense of presence group is used as between‐subjects factor (low vs. high sense of presence group), and electrode (Fz, Cz, POz) as within‐subjects factors. The table report analyses for each event‐related potential (ERP) component (mean area amplitude of each component as dependent variable), and separately for groups of low versus high sense of presence participants based on different questionnaire scores

**PQ**	**N1**	**MMN**	**SW1**	**SW2**
Group (1, 33)	1.17	.70	6.82*	5.08*
Electrode position (2, 66)	31.86^***^	23.74^***^	10.24**	3.10
Interaction	.63	.52	.15	.52

**SUS**	**N1**	**MMN**	**SW1**	**SW2**
Group (1, 34)	.53	.53	8.57**	4.97*
Electrode position (2, 68)	33.71^***^	24.43^***^	9.40**	1.87
Interaction	.20	.453	.391	.65

**MPS(sp)**	**N1**	**MMN**	**SW1**	**SW2**
Group (1, 29)	3.35^+^	1.59	11.63**	3.17^+^
Electrode position (2, 58)	31.67^***^	21.80^***^	9.04**	2.08
Interaction	2.76	.36	1.53	1.67

**MPS(se)**	**N1**	**MMN**	**SW1**	**SW2**
Group (1, 30)	2.02	3.36^+^	2.29	.27
Electrode position (2, 60)	28.03^***^	22.01^***^	8.07**	1.54
Interaction	.20	0.59	.18	0.04

^+^ *p* < .1.

* *p* < .05.

** *p* < .01.

^**^p < .001.

### 3.3 Association between the sense of presence subjective reports and brain activity

To explore the possibility of linear association between ERP components amplitude and sense of presence measured with a questionnaire, a series of Pearson's correlations were computed. Correlation analyses reduce the problems related to the grouping based on the median score of the questionnaire (e.g., the need to deal with participants having scores falling exactly on the group median). As previous analyses showed no interaction effects between the sense of presence group X electrode position, and to avoid multiple comparisons, instead of computing correlations for each one of the three electrodes separately, the ERP component amplitudes relative to the three studied electrodes (Fz, Cz, POz) were averaged together, creating a new variable for each ERP component (central cluster). Furthermore, the effect of sense of presence was shown not to greatly different between these electrode locations when analyzed independently (see Terkildsen & Makransky, 2019), and computing an average of a number of electrodes helps reducing possible noise that affects only some of the electrodes included in the cluster, increasing overall data quality.

**FIGURE 6:**
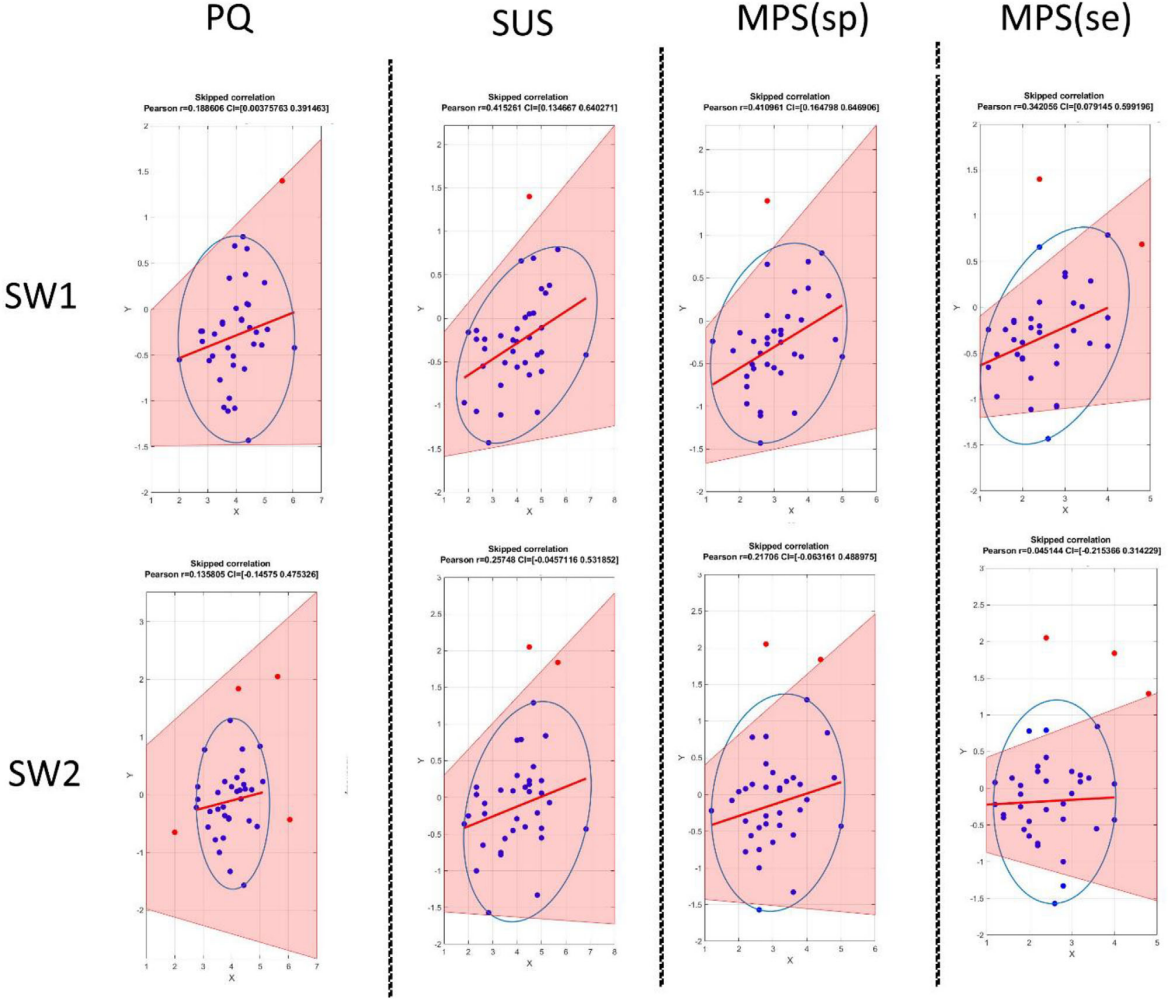
Robust (skipped) Pearson's correlations computed for the scores of every sense of PQ and the average values obtained in the SW1 and SW2 electrode clusters. Pearson's coefficients, and 95% confidence intervals (CIs) obtained by bootstrapping are shown on top of each one of the correlations scatter plots. The y‐axis shows the average values of the electrode clusters (μV), and the x‐axis shows the average questionnaire scores. Red lines are the regression lines, while the blue circle represents the distances from the orthogonal center of the data in which data points are accepted as valid. Red dots show data points considered outliers and removed before the correlation analysis. The blue dots are data points accepted as non‐outliers. The pink‐shaded areas shows the 95% bootstrapped CIs (see Pernet et al., 2013). All the correlation analyses for SW1 are statistically significant, while none of those for SW2 are significant

Consequently, individual values for N1, MMN, SW1, and SW2, were obtained for each participant. These values were then correlated with the subjective questionnaire scores, for the four different questionnaires measured. All the questionnaires showed statistically significant correlations with SW1 and SW2 ERP components amplitudes (see Table 3). Additional analyses computed Pearson's correlations using the robust correlations method (Pernet et al., 2013). Robust correlation analyses confirmed the association of the sense of PQs with the average amplitude of SW1; however, it showed that for SW2, the significance of previous analyses may have been driven by outliers (Figure 6).

**TABLE 3:** Correlations between ERP average amplitudes for ERP components of interest and sense of presence measured using the different questionnaires. One‐tailed p‐values are reported

	**N1**	**MMN**	**SW1**	**SW2**
PQ	.038	.106	.319*	.275*
SUS	.071	.022	.399**	.334*
MPS(sp)	.113	.047	.334*	.251^+^
MPS(se)	.193	.137	.366*	.280*

* *p* < .05.

** *p* < .01.

^**^p < .001.

^+^ *p* < .1.

## 4 DISCUSSION

The present study analyzed human brain electrophysiology (EEG) attempting to clarify if it is possible to establish psychophysiological correlates of sense of presence during immersive VR experience. At the same time, this study attempted to understand and clarify the heterogeneity of experimental results reported in previous investigations (Burns & Fairclough, 2015; Kober & Neuper, 2012; Terkildsen & Makransky, 2019). Several event‐related ERPs elicited by task‐irrelevant sounds in a classical auditory oddball paradigm were analyzed as possible physiological indexes for the subjective level of sense of presence as reported by the participants via questionnaires. The questionnaires evaluating the sense of presence used were four, the PQ, the SUS, and MPS(sp) and MPS(se) sub‐categories of the MPS questionnaire. In the experiment (primary task), participants were asked to partake in a VR roller‐coaster simulation. The VR scene was delivered using a modern customer‐oriented HMD (Oculus GO).

The participants did not have any explicit task to perform in the simulation (other than to explore the environment). Even if the participants were not instructed tasks or interaction with the VR environment, analyses of questionnaire data suggested participants experienced a good level of sense of presence (in line with previous studies using more interactive VR‐games, see e.g., Terkildsen & Makransky, 2019).

Based on results obtained from the questionnaires, participants were split into high and low sense of presence groups, following the same methodology used by previous investigations studying the electrophysiology of sense of presence (Burns & Fairclough, 2015; Kober & Neuper, 2012; Terkildsen & Makransky, 2019). To understand the role of different questionnaires to determine the participant grouping procedure, we used the scores of the four different questionnaires to establish four different participant splits. Successive analyses showed that the questionnaire used was relevant to define the participant's sense of presence group and depending on the questionnaires, various ERP components were found to be associated with the sense of presence. Late ERP components (slow waves) were found to be the most reliable electrophysiological index of sense of presence and were consistently associated with the scores of all the analyzed questionnaires.

### 4.1 Questionnaires evaluation

The subjective level of experienced sense of presence was evaluated using post‐experience questionnaires (PQ, SUS, MPS(sp), MPS(se)), and the median scores of these were used to assign participants into groups. The results of the questionnaires' scores correlated (moderate to high correlation). In the published literature is common that only one type of questionnaire or rating score is used to evaluate the sense of presence in VR, with the important exception of Kober and Neuper (2012), where PQ, SUS, and SFQ were used. However, recently, it has been shown that questionnaires may significantly differ from each other and often are based on different constructs and theoretical assumptions (Grassini & Laumann, 2020). The use of more than one questionnaire to evaluate the sense of presence in VR is relatively rare. PQ and SUS were previously found to be positively associated but not always the association was found to be statistically significant (as reported in Usoh et al., 2000). SUS and PQ were shown to be significantly positively correlated in more recent studies (Kober & Neuper, 2012, 2013). The MPS is a relatively new questionnaire, but due to its structure and its adaptability to various VR environments (as reported by the authors, see Makransky et al., 2017), it is lately being used and has been translated in different languages (see e.g., Berndt, et al., 2018; Makransky et al., 2019; Volkmann, et al., 2020). The MPS has been found to significantly correlate with the widely used igroup presence questionnaire (IPQ, Volkmann, et al., 2020); however, to our knowledge, there have not been attempts to study the association of the MPS to the PQ and SUS questionnaires as done in the present study. The results of the correlation analyses of the present study indicate that different sense of presence measures, even though based on different theories and differing in structure, do generally correlate (at least moderately), in line with some previous studies (Bouchard et al., 2008; Kober & Neuper, 2012).

Nevertheless, when we used the questionnaires’ scores to divide the participants into the high and low sense of presence groups based on overall median scores, as done in previous studies (Burns & Fairclough, 2015; Kober & Neuper, 2012; Terkildsen & Makransky, 2019), more than 50% of participants changed their group (high, low, and median) at least once depending on the questionnaire the split was based.

### 4.2 Electrophysiology of sense of presence

Event‐related brain responses elicited by the task‐irrelevant tones during the VR roller‐coaster experience were analyzed to understand if different ERP components could serve as an indicator of the subjective sense of presence. From the inspection of the waveforms (Figures 3 and 4), the low sense of presence participants’ group showed a relative increase in all the components of interest (N1, MMN, SW1, and SW2). The trend of these differences is based on Figure 5. Scalp topographies were more pronounced over frontal brain areas for the early components (N1 and MMN) and over central brain areas for later ones (SW1 and SW2). However, statistical analyses did not show an interaction between the different groups and electrodes position. From visual inspection of the brain signal distribution in the scalp topographies (Figure 5) is possible to deduce that the components divided in the analyses as SW1 and SW2 may be related to the same cognitive phenomena, and they might be considered as a singular component. This is confirmed from the similar results obtained for SW1 and SW2 in relation to the sense of presence.

When data were analyzed with the grouping strategy, statistical differences in the late ERP components SW1 and SW2 were found between the two groups when all the questionnaire scores were considered, except for MPS(se). The difference in results between the different questionnaires may be due to the different construct of sense of presence analyzed. While PQ, SUS, and MPS(sp) evaluate spatial/physical presence (Lee, 2003; Witmer & Singer, 1998), the MPS(se) estimate the different construct of self‐presence (Lee, 2004b).

Statistical differences in the early ERP components N1 and MMN were not found in PQ and SUS questionnaires grouping. However, when grouping was established on MPS sub‐categories, the low sense of presence group showed a more pronounced N1 component, compared to the high sense of presence group (for the MPS(sp)), and the same was shown for the MMN component (for the MPS(se)). These differences were not statistically significant but approached significance.

Further correlation analyses were carried on, aiming to understand a possible linear association between the mean amplitude of the components and subjective sense of presence. For those analyses, participants were not grouped. SW1 and SW2 components showed a generally moderate linear association with all the questionnaires, and the association was statistically significant in all the correlations. Instead, none of the association analyses with early components (N1, MMN) and questionnaire results showed to be statistically significant. These findings support the idea that even if highly correlated, different questionnaires could evaluate somewhat different sorts of sense of presence, especially when the grouping strategy based on post hoc questionnaire results is used (Burns & Fairclough, 2015; Kober & Neuper, 2012; Terkildsen & Makransky, 2019). Furthermore, the correlation analyses showed the association between late components SW1 and SW2, and sense of presence scores was found to be linear and consistent among the different questionnaires (with the sole exception of SW2 (MPS(sp))). For the early ERP components, the relationship was found weakly associated, and it is likely to be a discrete difference between the participants’ groups and/or that participants’ with scores close to the median were responsible for the differences between groups individuated in previous analyses (for MPS(sp) and MPS(se)). The linear association was shown to be robust against extreme values in the sample for the SW1 component and all the questionnaires analyses but not for SW2. The association between questionnaires and SW2 identified by standard Pearson's correlation analyses was shown by robust analyses to be driven by outliers. It is possible that the late SW2 components, peaking on a later processing stage, compared to the earlier SW1, would contain more activity non‐related to the attentional processes toward the physical stimulus (the tone), and in general, more artifacts non‐related to the stimulation, compared to SW1.

It has been proposed that amplitudes of SWs may be associated with the level of cognitive stimuli processing. Earlier studies have shown that late components as the studied SW1 and SW2 may be related to mental workload and has been proposed that SWs amplitude may indicate the cognitive resource allocation to information processing (Rösler et al., 1997). In an auditory oddball task study (Squires et al., 1975), it has been found that slow waves prompted by the infrequent tones peaked at 400–500 ms after stimulus onset. The magnitude (amplitude) of the slow waves was positively modulated (more negative waves) when the participants were asked to discriminate the tones, compared to a condition where they were asked to ignore them. These results were interpreted as an association of the magnitude of the negative amplitude of the slow waves with the attention shift (Kok, 1997; Squires et al., 1975).

In the present investigation, the low sense of presence group may have perceived the task‐irrelevant tones as more relevant, compared to the group of participants who were feeling more present. In turn, the more pronounced attentional processes toward the auditory stimuli may be reflected in the increased (more negative) amplitude in the SWs, when the low sense of presence group was compared with the high sense of presence group, in line with the attentional allocation theories aforementioned (Kok, 1997; Squires et al., 1975).

However, the study of Terkildsen and Makransky (2019) did not support the hypothesis that the SW components may be associated with the sense of presence. In their study (based on the same design of Burns & Fairclough, 2015; Kober & Neuper, 2012), they found no difference in SW for the high and low sense of presence groups, reporting only a weak relationship between SW1 and the spatial presence score of the MPS. Instead, Terkildsen and Makransky (2019) found that N1 and MMN amplitudes were significantly different in the high sense of presence group, compared to the low one, and they interpreted such results as in line with the attention‐based theoretical operationalization of presence of Wirth et al. (2007). Such theoretical approach claims for a significant “devotion of mental capacities to the media product” (Wirth et al., 2007, p. 497) and would justify, according to Terkildsen and Makransky (2019), the fact that a high sense of presence would deplete early and automatic attentional processes toward stimuli outside the VR.

According to Terkildsen and Makransky (2019), the studies of Burns and Fairclough (2015)–that reports as well an association between SWs and sense of presence–may have found an association of SWs with challenge‐based immersion and not directly an association between SWs and sense of presence. As Burns and Fairclough (2015) used different game difficulties in their VR condition, it is possible that changes in challenges may have modulated attentional processes toward the infrequent tones of the secondary task.

Terkildsen and Makransky (2019) proposed that a similar rationale could be applied to the results reported by Kober and Neuper (2012). It is possible that the challenge of goal‐oriented navigation tasks in the city VR simulation of Kober and Neuper (2012) significantly influenced the sense of presence of the participants. Therefore, the participants in Kober and Neuper (2012) may have been divided into the high and low sense of presence groups based on challenge‐based immersion consequential from individual participants’ differences in perceiving the difficulty of the navigation task and not based on interpersonal differences in users’ sense of presence.

Nevertheless, the VR environment used in our study was chosen purposefully for not promoting challenge‐based immersion (as the participants do not have to perform any task in the VR environment), but our results are only partially in line with those of Terkildsen and Makransky (2019). Therefore, the interpretation of previous results (Burns & Fairclough, 2015; Kober & Neuper, 2012) as proposed by Terkildsen and Makransky (2019) is not supported by the results shown in the present study.

The heterogeneity of results obtained with the division in groups of the sample showed how this strategy for data analysis could be problematic, especially in EEG studies where the number of participants is generally not very high. The result of the present study shows that correlational analysis may provide more robust results.

### 4.3 Theoretical implications

In our study, the late components amplitudes (SWs) elicited by the deviant tones were shown to be decreased for the high sense of presence group, compared to the low sense of presence group, as their involvement in VR captured most of their cognitive capacities. The low sense of presence group, as it was less involved in the VR, had more attentional resources available to process the deviant tones, and therefore showed an increased (more negative) SWs amplitude. The SW modulation was shown to be reliable for every grouping strategy using a different questionnaire.

The other ERP components analyzed (N1, MMN) did not consistently show to differ between the two groups and were found to correlate with the sense of presence only when the groups were estimated using the MPS questionnaires. The N1 component is associated with the allocation of early perceptual processes (e.g., encoding of elementary stimulus features, see e.g., Kok, 1997; Näätänen et al., 2011). The lack of differences between the high and low presence groups in N1 component may be due to the sensory processing of low‐level stimulus features that was probably very similar between both groups, as the physical features of the stimulation were the same (as proposed in Kober & Neuper, 2012), though the N1 component was found to be related to the sense of presence when grouping was based on the MPS(sp) questionnaire. The MMN component reflected automatic processing, and it is elicited even in the absence of attention (Näätänen et al., 2011). This component was found to be related to the sense of presence but only when grouping was based on the MPS(se) questionnaire.

These latter findings may suggest that attentional processes related to early low‐level stimuli features and automatic sensory processes may be sensitive to some features of the sense of presence and that those features may be assessed by some questionnaires but not to others.

However, the study by Terkildsen and Makransky (2019) found only N1 and MMN to be associated with the sense of presence, while their results for the SWs were inconsistent. It is possible that their experimental setup promotes early and automatic attentional processes more than those used in the present article and earlier ones. The Terkildsen and Makransky (2019) VR environment is the only one among the ones previously used (the present study; Burns & Fairclough, 2015; Kober & Neuper, 2012) that strongly promotes emotional states. Being an immersive horror‐themed videogame, the VR used in Terkildsen and Makransky (2019) may have induced a reasonably high level of fear. The feeling of fear and threat has been studied in relationship with early and automatic attentional processes (see e.g., Grassini et al., 2019a, 2021b), and it is possible that a state of visually induced fear may promote different attentional processes in other sensory modalities (early auditory attention in the case of Terkildsen & Makransky, 2019). The activation of earlier attentional processes may then deplete the attentional resources for later (consciously controlled) attentional processes (mirrored in the brain activity in SWs). The allocation of early attentional resources (associated with N1 and MMN) and the allocation of later ones (SWs) might be mutually moderating in the framework of a dual‐task design as the one hereby used, as the limited attentional resources available would be allocated–depending on the perceptive context–only toward early or later perceptual processes. The allocation of visual attention toward early ERP components depending on visualization context was recently suggested by an electrophysiological study on perception threatening stimuli (Grassini et al., 2019b). This hypothesis would explain how earlier studies that have used this experimental paradigm have found the sense of presence to be associated with both early and late ERP components of perception. It is possible that our VR environment–a roller coaster ride–induces some degree of emotional arousal or fear to some participants. Therefore, our VR environment may have modulated to some degree the participants' attention–or only for those participants experiencing the highest level of emotions–toward earlier attentional processes. Admittedly, this explanation is only speculative as we do not have information about the participant's subjective emotional state during the VR experience.

A further possibility to explain the heterogeneity of results in previous studies, as already suggested earlier in the manuscript, is related to the sensitivity of the various questionnaires to different aspects of sense of presence. Terkildsen and Makransky's (2019) study used the MPS questionnaire, and we have shown in the present study that such questionnaire (at least for the analyzed physical and self sub‐components) seems to be associated more with early N1 and MMN, compared to the other questionnaires. However, our findings are not conclusive and only partially support the latter hypothesis, as not both sub‐categories of the MPS questionnaire converge toward the same results as presented by Terkildsen and Makransky (2019). The two hypotheses presented above are not mutually exclusive, and a combination of different and uncontrolled factors may have driven the heterogeneity of the results reported in previous studies.

### 4.4 Limitations

The present study presents several limitations. Other components of the ERPs were not analyzed in the present study, and only a few were selected a priori for analysis. The P3 component (300–600 ms from stimulus onset) elicited by deviant tones has sometimes been analyzed in the context of the auditory oddball paradigm, and it is thought to be related to post‐perceptual cognitive processes as a response to specific auditory stimuli (Donchin et al., 1986; Kok, 2001; Näätänen et al., 2011; Polich, 2007). In the context of the present study, the tones were task‐irrelevant and did not require any action or evaluation from the participants. Hence, the analyses of the P3 component were not theoretically supported. Furthermore, none of the previous studies using similar paradigms for the study of the sense of presence analyzed P3 (Burns & Fairclough, 2015; Kober & Neuper, 2012; Terkildsen & Makransky, 2019).

To our knowledge, the present study is the first that has used a dual‐task auditory oddball paradigm and EEG to physiologically investigate the sense of presence using a highly immersive technology to mediate the VR experience (HMD). Hence, our findings, even though in line with these reported by the scientific literature that used traditional systems (e.g., desktop monitors) to mediate the VR environment, may not be completely comparable. Even though increasingly popular (see e.g., applications as the ones presented in Cattan et al., 2019; Vourvopoulos et al., 2019), the use of EEG together with HMDs may be problematic due to interferences between the systems. However, recent analyses (see e.g., Hertweck, et al., 2019; Tauscher et al., 2019) showed that the problems of combining these two systems are not substantial and EEG data quality is minimally affected.

A further limitation is related to the questionnaire instruments we used. The SUS questionnaire is the only one where all the questionnaire items were coherent and applicable for our VR environment. This was due to the type of VR environment that we used, as the participants were not asked to perform any specific tasks but instead only experience the VR environment. The number of the items in the PQ was about halved due to the need for adapting the questionnaire to our VR environment (e.g., interactions with items, the possibility of movements, etc.). This may have impaired the validity of the inventory to measure the construct. As our environment did not contain social interactions or social components, the social sub‐component of the MPS questionnaire was also eliminated as irrelevant.

An important limitation of the interpretation of the data based on the median split is that the median split resulted in quite even (PQ, SUS) or rather uneven (MPS) subgroups used for the ERP comparison. This is a limitation that affects all the studies where a median split is used, as several participants may show results right in the median score, and therefore they need to be removed or assigned to one of the groups. Future studies that plan to utilize the median split analysis method using the experimental design described in the present article may attempt to mitigate the problem of data bias by recording, analyzing, and comparing the ERPs in response to the pre‐VR exposure auditory oddball. Such analysis would help to clarify whether observed differences in the ERPs are truly due to the experience of the sense of presence or simply due to pre‐existing differences between the groups. Unfortunately, in the present study–that was designed following the description of the experimental paradigm proposed in Terkildsen & Makransky, 2019), pre‐VR exposure EEG was not recorded; therefore, it is not possible to assess if such bias exists in the analysis where two groups were established based on median splits. The analysis of pre‐VR ERPs may also help to understand whether the reduced ERP amplitudes might truly be related to the sense of presence or whether they rather reflect an individual trait variable (e.g., how easily participants could be distracted by task‐unrelated auditory stimuli). This possible confound has not been studied in any of the previous studies that have used this experimental paradigm and deserves future clarification.

Due to the particular nature of the VR environment we used in this experiment, our results are difficult to generalize to environments where movements and interactions are required from the user. Such aspect also reduces the ecological validity of our study in respect to videogames or training and teaching‐oriented VRs, where actions are requested from the user.

Finally, the role of simulator sickness and other types of discomfort mediated by immersive HMDs (see e.g., Grassini & Laumann, 2021) were not investigated or analyzed in the present study. These phenomena may, however, have an influence on attentional and cognitive processes, and therefore they could represent a confounding factor. In the present study, we assumed that feelings of discomfort during the use of the HMDs were randomly distributed among participants with a different subjective experience of the sense of presence.

### 4.5 Future directions

One of the possible limitations of the present study is the theoretical a priori selection of cognitive phenomena (and therefore ERP correlates) of interest. However, a data‐driven approach to the electrophysiological data (see e.g., Grassini et al., 2019b; Langford, 2016; Wynn et al., 2019) may be worth in the context of the present state of the research to confirm the topography and latency of potential ERP correlates of the sense of presence.

Even though using late ERP components as objective indexes to assess the sense of presence in VR seem to be promising, future studies should attempt to replicate our findings. An interesting perspective is represented on understanding how induced emotions in VR (as in Terkildsen & Makransky, 2019) may be able to modulate attentional processes toward stimuli in the real environment. The interaction between experience in VR and simultaneous experience in the real world should be further explored, and it could be exploited in practical applications of highly immersive VR technology.

The MPS (Makransky et al., 2017) questionnaire and its sub‐components are a promising tool for investigating various aspects of sense of presence. However, it may be possible that the MPS questionnaire spatial presence component may investigate aspects of spatial presence that are different from those investigated by the PQ and SUS. Such a possibility may be worthy of further investigations using behavioral quantitative and qualitative methods. We believe that finding a reliable and easily quantifiable index of user experience (as the sense of presence may represent) might be important for practical application as the evaluation of hardware and software.

## 5 CONCLUSION

The late ERP components elicited by VR‐irrelevant tones were found to be related to the subjectively reported sense of presence via post‐exposure questionnaire. This effect was shown to be consistent among questionnaires evaluating physical/spatial presence. Some questionnaires (MPS) may be more sensitive to aspects of the physiology of sense of presence that is reflected in early attentional activities. Furthermore, we show that the method of participants grouping based on the median score of the questionnaire–as used in several previous studies–may critically affect the results and that participants’ grouping based on different questionnaires–even when these questionnaires correlate–may be accountable for the heterogeneous results reported in the literature. Different questionnaires may be indicated to explore different aspects of sense of presence, and therefore are associated with different physiological phenomena. However, our reported findings in this respect are inconclusive. The late, negative slow waves recorded over the central brain areas showed a good linear association with the questionnaires used, and this association was found to be robust to outlier values for the SW1 component. Nevertheless, no linear association was found for subjective reports of sense of presence and early brain activity. Taken together, our results suggest that the late ERP components of the EEG, recorded from the central brain area, are (among the ones investigated) the most reliable electrophysiological correlate of the subjective sense of presence.

## AUTHOR CONTRIBUTIONS

Simone Grassini was responsible for the study idea, the experimental design, the management of the lab work, the data analysis, the training of junior lab. assistants, and for writing the present manuscript. Karin Laumann provided funding for the project, gave feedbacks at every stage of the study, and commented on earlier drafts of the present manuscript. Virginia de Martin Topranin and Simone Grassini assisted in the development of the experimental design, had the responsibility to test the participants in the laboratory, and commented on earlier drafts of the manuscript. All the authors have read and approved the final version of the article.

### PEER REVIEW

The peer review history for this article is available at https://publons.com/publon/10.1002/brb3.2269.

## Supporting information

Supporting information

## Data Availability

The data used for statistical analyses is openly available at https://doi.org/10.5281/zenodo.4686925.
