# Supplementary material for: Using electrophysiological measures to evaluate the sense of presence in immersive virtual environments: An event‐related potential study
Source: Brain Behav. 2021 Jun 26;11(8):e2269. doi: 10.1002/brb3.2269 (PMC8413821; doi:10.1002/brb3.2269)
Supplement: Supplementary file 1 — Supporting information [file BRB3-11-e2269-s001.pdf]

Supplementary data

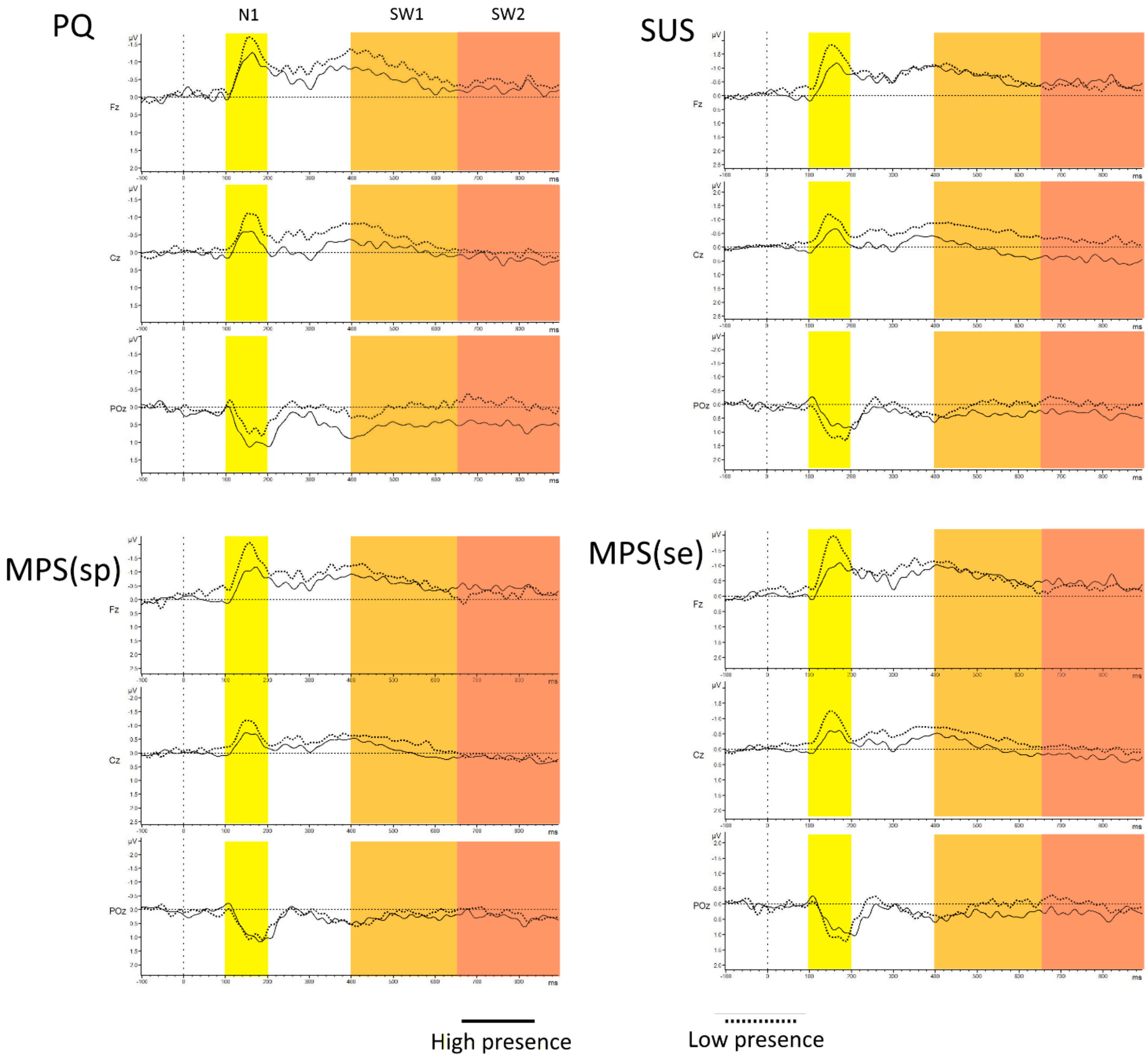

Supplementary Figure 1. Grand average ERPs for deviant tones recorded in electrodes Fz, Cz and POz for both presence groups. The presence groups estimated based on different questionnaires are presented separately. The highlighted time-windows indicate the latency windows of interest; N1 (100–200 ms), SW1 (400–650 ms), SW2 (650–900 ms) from the deviant tone onset. Baseline activity is shown (-100 ms to 0 [event onset]).

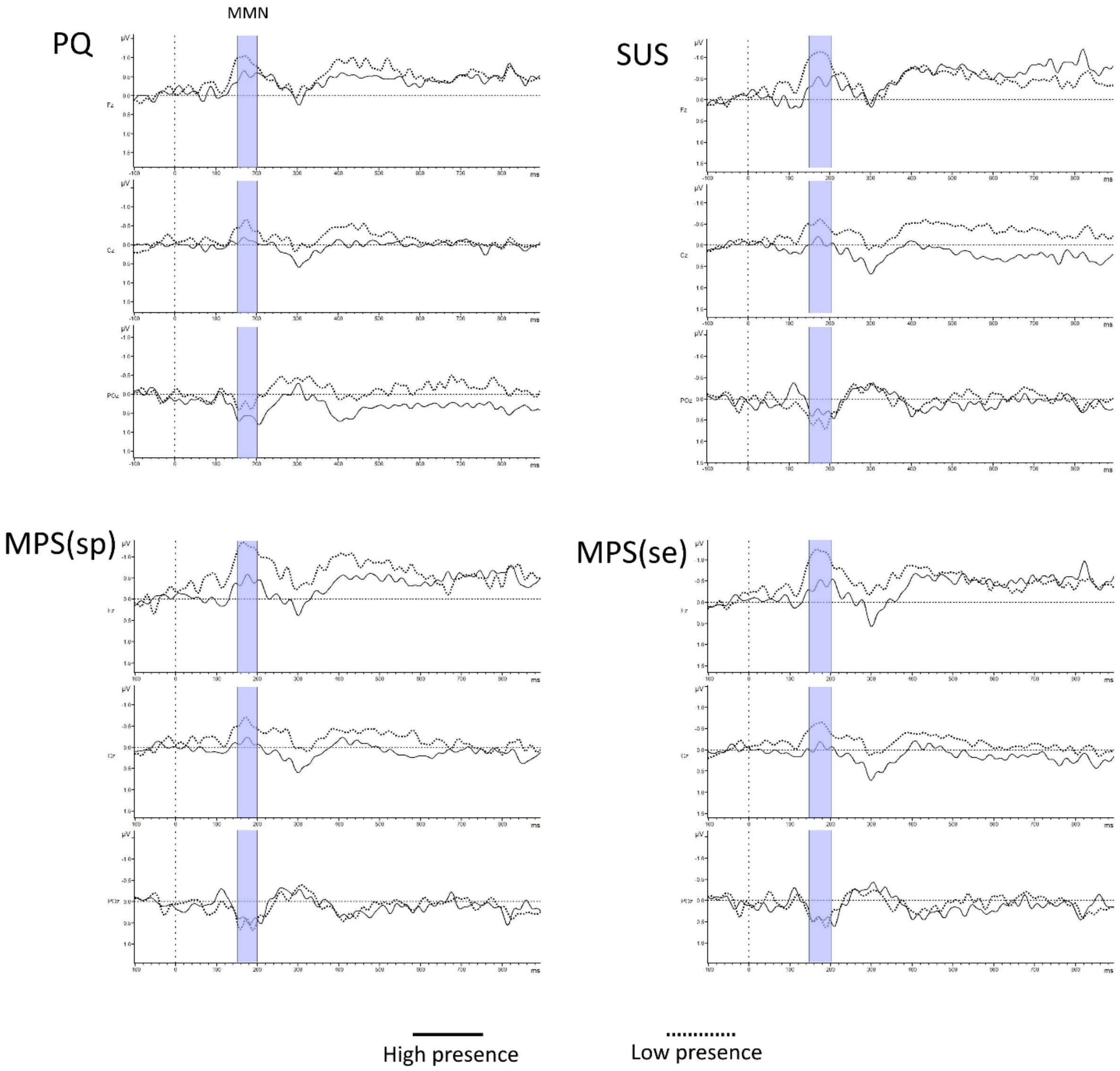

Supplementary Figure 2. Grand average ERPs with MMN (150-200 ms) component highlighted. The waves were computed subtracting brain activity time-locked with frequent tones from the one time-locked with deviant tones, separately for high-and-low presence groups, established using different questionnaires on sense of presence. Baseline activity is shown (-100 ms to 0 [event onset]).
